# New species of *Kuqaia* from the Lower Jurassic of Sweden indicates a possible water flea (Crustacea: Branchiopoda) affinity

**DOI:** 10.1371/journal.pone.0282247

**Published:** 2023-06-07

**Authors:** Jungang Peng, Sam M. Slater, Stephen McLoughlin, Vivi Vajda

**Affiliations:** 1 State Key Laboratory of Palaeobiology and Stratigraphy, Nanjing Institute of Geology and Palaeontology and Center for Excellence in Life and Paleoenvironment, Chinese Academy of Sciences, Nanjing, China; 2 Department of Palaeobiology, Swedish Museum of Natural History, Stockholm, Sweden; Peking University, CHINA

## Abstract

The enigmatic acid-resistant mesofossil genus *Kuqaia* is emended, a new species (*Kuqaia scanicus*) is instituted, and three established species are described from the Lower Jurassic (lower Pliensbachian) of the Kävlinge BH-928 core, in southern Sweden. *Kuqaia* has a distribution across the middle northern latitudes of Pangaea and is restricted to Lower to lower Middle Jurassic strata. Morphological characters support *Kuqaia* being the ephippia (resting egg/embryo cases) of Cladocera (Crustacea: Branchiopoda), and a probable early stem-group taxon of the *Daphnia* lineage. The paleoecology of the small planktonic crustaceans indicate purely fresh-water environments, such as lakes or ponds, all occurrences being in continental deposits, and the *Kuqaia* specimens possibly represent dry-season resting eggs. Chemical analyses of these and similar fossils, and of extant invertebrate eggs and egg cases are recommended to improve resolution of the biological affiliations of such mesofossil groups.

## Introduction

The end-Triassic mass extinction (ETME) is one of the ‘Big Five’ biotic crises within the Phanerozoic, according to marine and terrestrial fossil records (e.g. [1, 2]). The ETME witnessed the demise of many lineages of marine animals (e.g., conodonts, conulariids and several orders of brachiopods; [3] and references therein) and key groups of terrestrial plants (e.g., Peltaspermaceae; [4]), and was followed by the emergence of a range of novel biological forms. In the oceans, Plesiosauria originated at *c*. 200 Ma, and represented a long-lived radiation of secondarily marine, non-mammalian tetrapods [5]; Cyrtocrinids appeared abruptly in the deep sea and radiated before the Sinemurian [6]; ichthyosaurs and ammonoids also underwent radiations in the aftermath of the ETME [7, 8]. On land, dinosaurs survived the ETME, and radiated rapidly into a broad range of ecological niches [9, 10]. The terrestrial vegetation also experienced marked changes with, e.g., Caytoniales, Pentoxylales, Leptostrobales, modern groups of conifers, and new groups of Bennettitales diversifying in the aftermath of the ETME [11–13].

Given these striking changes on land and in the oceans, we would expect a surge in similar novel forms among the heterotrophic microbiota, especially since there were notable radiations among phytoplankton groups during the post-ETME interval [14, 15]. Thus far, only marine carbonate- and silica-shelled protists have yielded evidence of this re-radiation [16, 17]. Here we provide evidence of the diversification of a novel non-calcareous and non-siliceous heterotrophic mesofossil group—the enigmatic *Kuqaia* Li, 1993.

*Kuqaia* is a morphologically distinct palynomorph taxon characterized by a HF-resistant, bilateral symmetrical, ornamented ‘shell’ bearing a caudal spine [18]. Based on shell ornamentation, six species have been described so far, i.e., *Kuqaia concentrica* Li, 1993, *K*. *cucuma* (Yang & Sun, 1987) Cui *et al*., 2004, *K*. *quadrata* Li, 1993, *K*. *radiata* Li, 1993, *K*. *yangii* Cui *et al*., 2004 and *K*. *yanqiensis* Cui *et al*., 2004. *Kuqaia* has been recorded from China [18–27], offshore mid-Norway [28], and the northern part of the North Sea [29].

*Kuqaia* has been used as an index fossil for Lower and Middle Jurassic strata in the Tarim, Yanqi, Junggar and Qaidam basins, northwestern China [18, 20–27]. Its occurrences, within Hettangian to Pliensbachian successions of the mid-Norwegian Shelf [28], and in Aalenian successions of the northern North Sea [29], are consistent with its restricted (Lower–Middle Jurassic) stratigraphic ranges recorded from China.

In this study, specimens from the Kävlinge bore core BH-928 in southern Sweden are investigated. We describe and identify several specimens of *Kuqaia* as belonging to three known species, and we describe one new species based on a well-preserved specimen with distinctive morphological characters. We review the palaeogeographic and biostratigraphic distributions of *Kuqaia*, highlight its utility in biostratigraphy, and evaluate its biological affiliation.

### Geological setting

**Fig 1:**
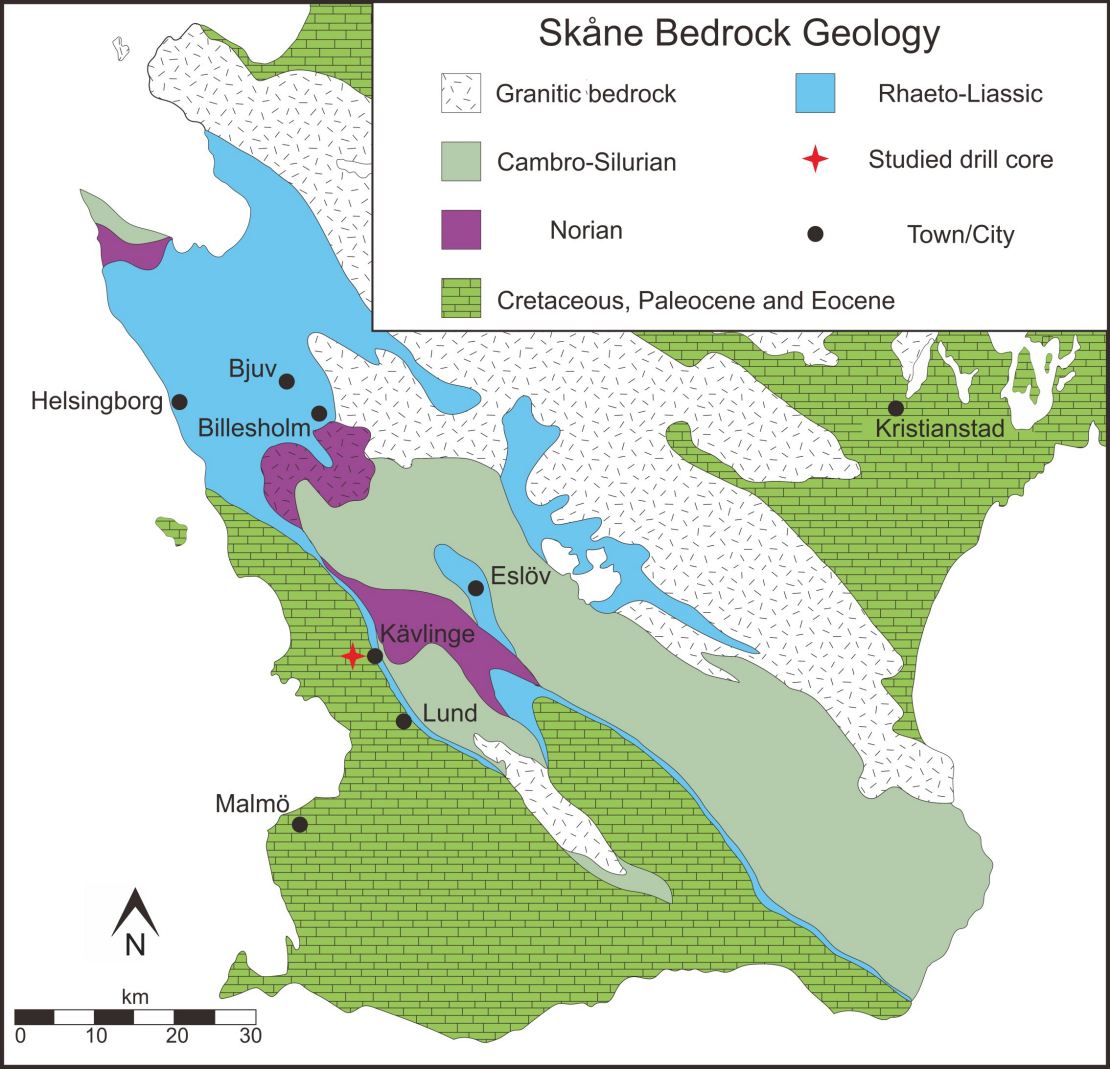
Geological map of Skåne (southern Sweden), showing the location of the studied drill core Kävlinge BH-928, modified after [33].

Specimens studied here derive from the Kävlinge BH-928 bore core, which was drilled in the valley of the Kävlinge River, to the west of Kävlinge village in Skåne, southern Sweden (Fig 1). The sampled succession, which is included in the Katslösa Member, Rya Formation [30], encompasses the interval from 83.25 m to 28.75 m in the core. The lower portion, between 83.25 m and 54 m, is represented by grey-green sand and sandstones that are locally rich in oolitic chamosite [31], suggesting low-energy marine depositional environments chiefly below wave base [32]. This lower portion contains a moderately rich foraminiferal assemblage dated to the ‘upper Liassic γ’ (lower Pliensbachian [31]). A regression occurred during the deposition of the Katslösa Member, and the upper portion, above 54 m, is devoid of foraminifera but yields megaspores and the *Kuqaia* mesofossils identified here.

## Material and methods

Nine mesofossil specimens (NRM X12700–NRM X12708) were picked from residues originally collected for studies of foraminifera by Dr Erik Norling in the 1960s [31]. Although Norling identified the foraminifera, other miscellaneous fossils, including megaspores, *Kuqaia* and fossils of unknown affinity, were not described. These are all stored in the microfossil collections of the Department of Palaeobiology, Swedish Museum of Natural History (NRM). The *Kuqaia* specimens are stored in four covered sample trays, collected from the following sample depths; 57.00–56.50 m (one specimen), 46.97–46.01 m (five specimens), 42.25–41.15 m (two specimens) and 40.40–39.60 m (one specimen). Specimens were studied using reflected light microscopy (Olympus SZX10), transmitted and fluorescence light microscopy (Olympus BX51 with UV light) and scanning electron microscopy (ESEM FEI Quanta FEG 650, NRM). For details of microscopy methods, see [33].

### Systematic palaeontology

The descriptive terminology for *Kuqaia* varies between publications, some advocating the terminology used for ephippia (resting eggs) of cladocerans [18, 25], whereas others have employed the morphological terminology for palynomorphs [20]. Here we follow the terminology for cladoceran ephippia applied previously to Chinese examples [18]. Although cladoceran ephippia strictly represent protective casings enclosing one or more eggs or developing embryos, they have a surperficially saddle- or shell-like morphology and, traditionally, the main body of the ephippium has been termed the ‘shell’. Specimens have a short vertical and long transverse axis. The lower side with a line of dehiscence is termed the venter, and the opposite side is the dorsum; the terminus bearing spine(s) or long projections is designated the posterior (caudal) end; the opposite end representing the anterior. The ‘postventral margin’ refers to parts of the ‘shell’ between the centre of the ventral margin and the caudal spine. The ‘back’ represents the line on the dorsum, which divides the ‘shell’ into two symmetrical parts [18]. We introduce the term ‘peduncle’ to describe each of the long terminal projections of *Kuqaia scanicus* sp. nov., since this term was used to describe the slender appendages extending from the ventral margin of the ephippium posterior in cladocerans [34]. Measured length represents the maximum distance from the anterior to posterior (excluding the peduncle), and width represents the maximum distance from venter to dorsum.

Genus *Kuqaia* Li, 1993 emended.

1993 *Kuqaia* Li, pp. 72, 74.

*Type species*. *Kuqaia quadrata* Li, 1993.

*Emended diagnosis*. Shell single-layered, bilaterally symmetrical, splitting along venter. Shell surface ornamented with a series of concentric and/or radial ridges. Concentric and radial ridges initiate on the ventral side. Caudal end tapered, acute. Peduncles variably present on the postventral sides.

**Fig 2:**
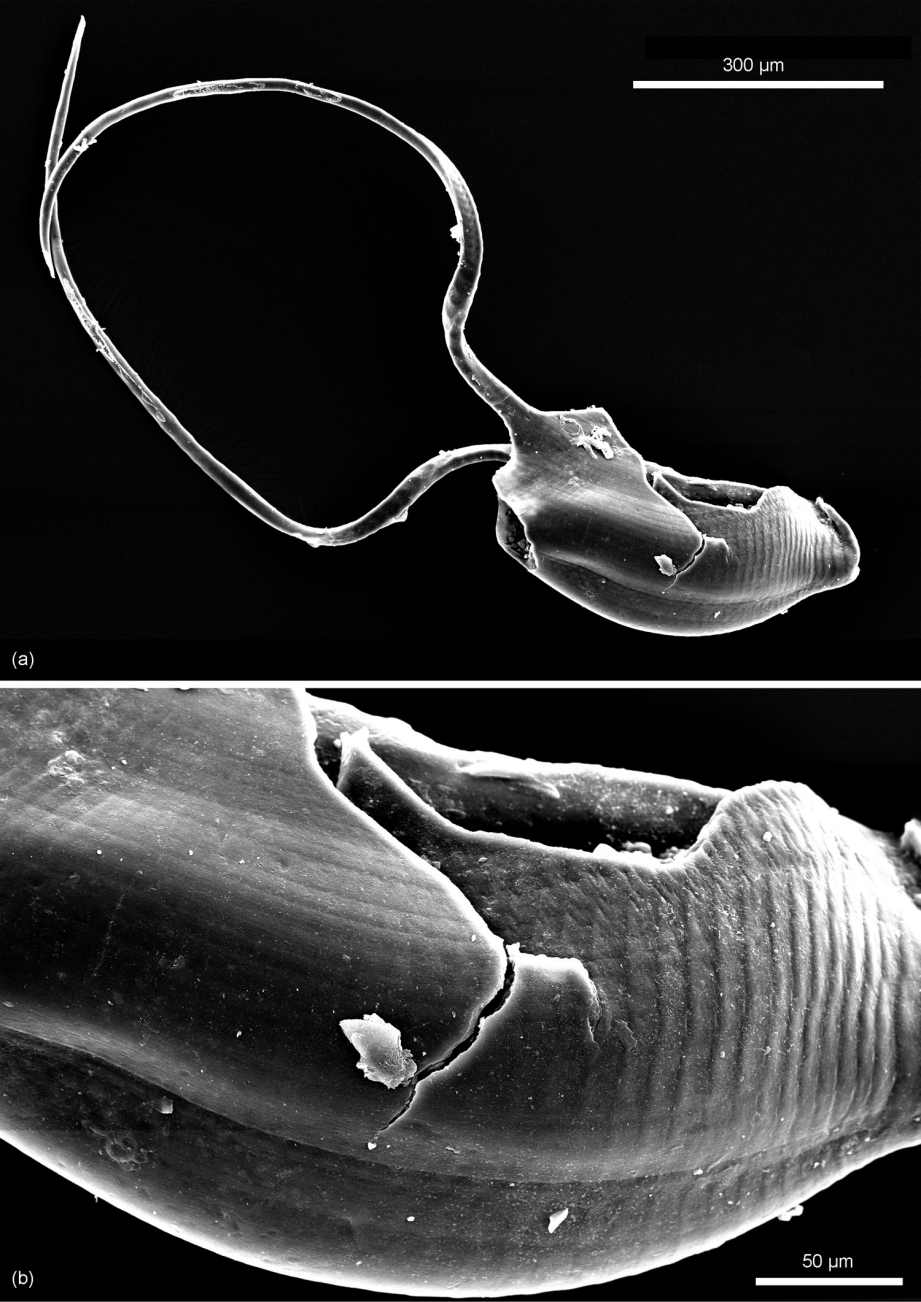
*Kuqaia scanicus* sp.nov. Holotype, NRM X12700; Kävlinge BH-928 core depth 40.40–39.60 m. (a) Scanning electron micrograph of whole specimen at low magnification. (b) Enlargement (SEM image), showing surface ornamentation in central part.

**Fig 3:**
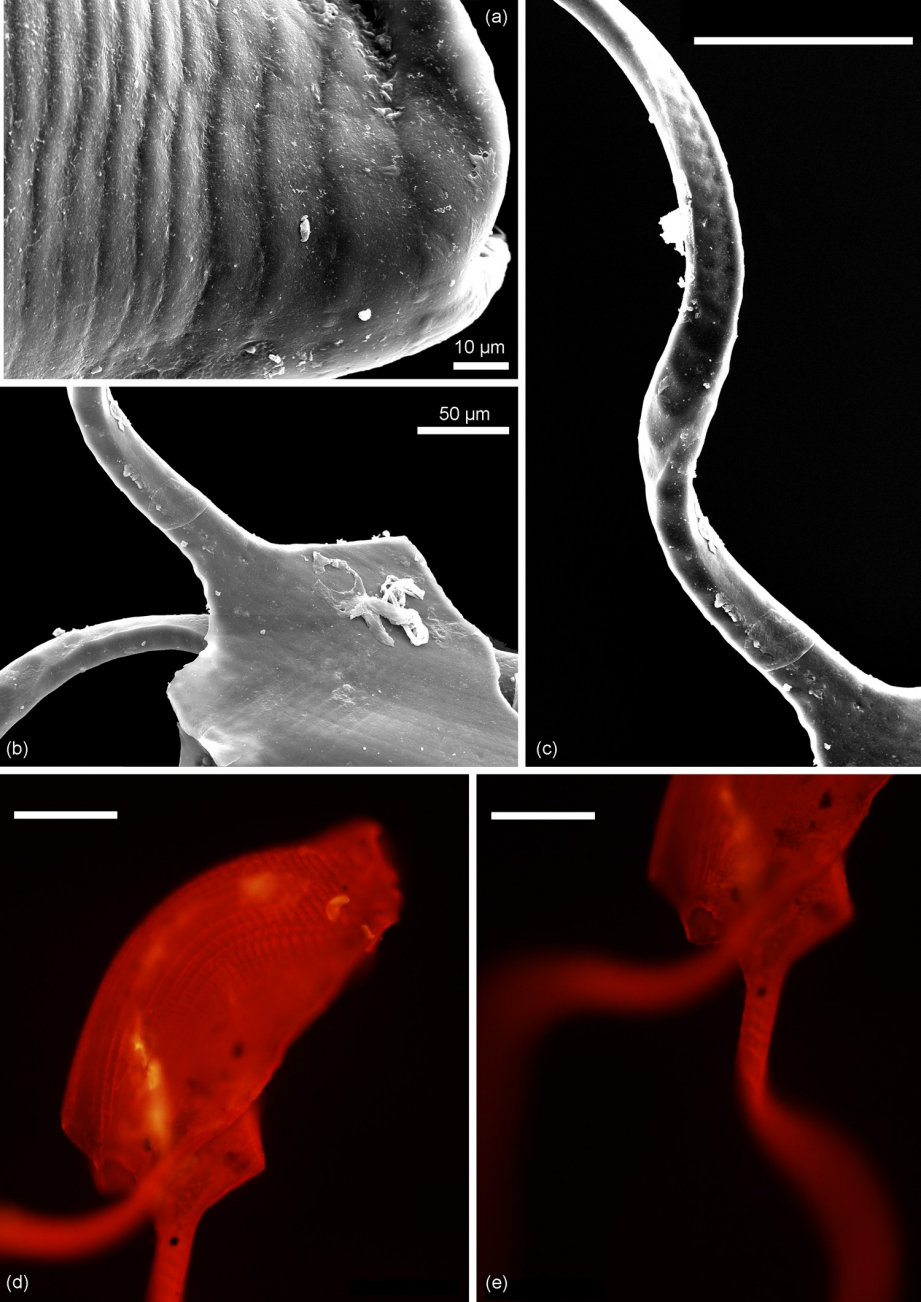
Enlargement of the holotype (NRM X12700) of *Kuqaia scanicus* sp. nov. Kävlinge BH-928 core depth 40.40–39.60 m. Scale bars = 100 μm, unless otherwise stated. (a) Well-developed radial ridges at the anterior (SEM image). (b) Weakly defined ridges at the posterior (SEM image). (c) Weak, parallel and transverse ridges on peduncles (SEM image). (d) Complex surface ornament revealed in UV-fluorescence microscopy. (e) Weakly defined ridges on peduncles evident in fluorescence microscopy.

*Remarks*. The diagnosis of *Kuqaia* is here emended to encompass characters of *Kuqaia scanicus* sp. nov., i.e., the presence of two lateral peduncles at the caudal end (Figs 2 and 3).

**Fig 4:**
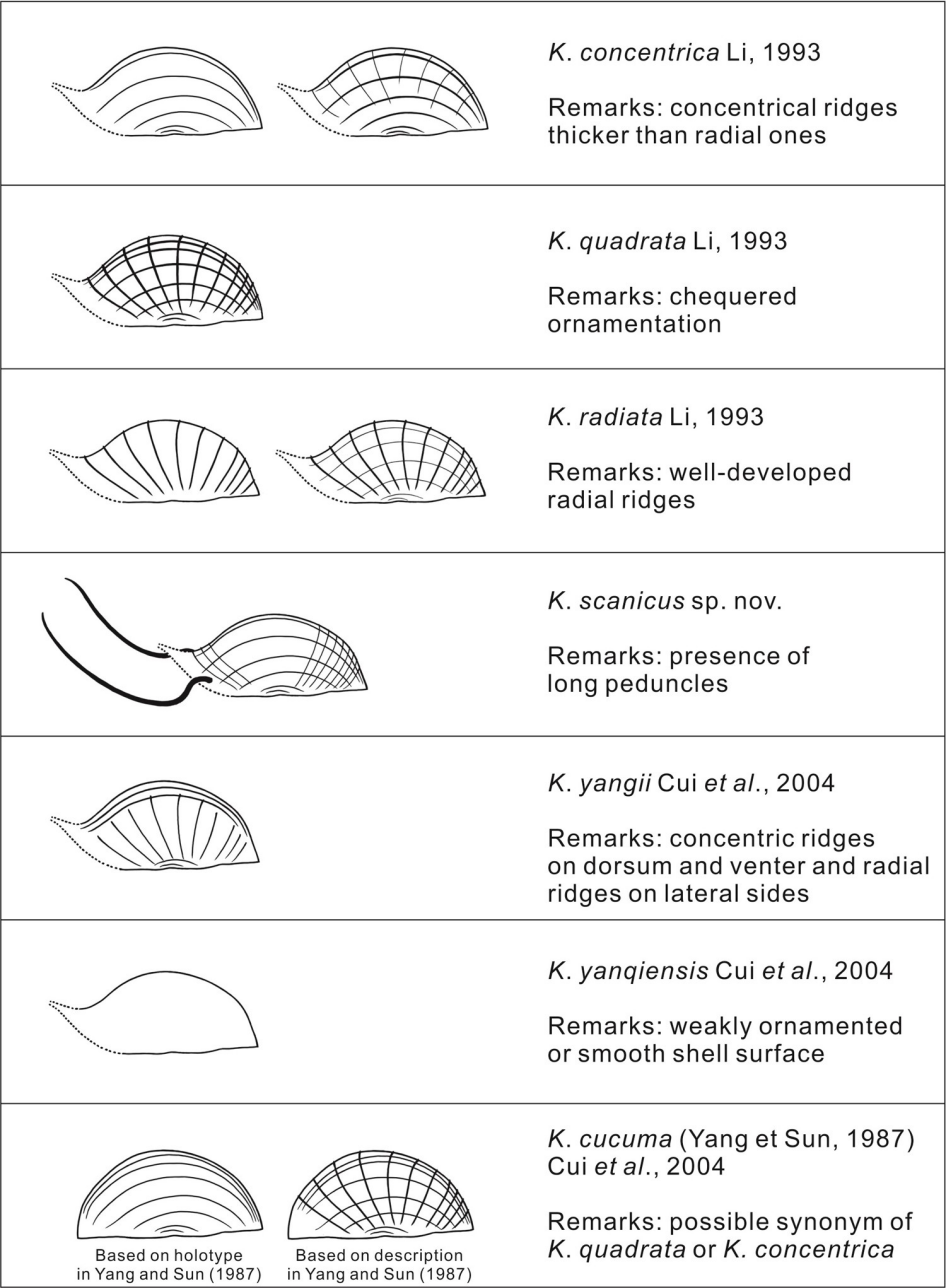
Line drawings showing the morphological characteristics of *Kuqaia* species. Note that transitional types were indicated for *Kuqaia concentrica* and *K*. *radiata*.

Three species of *Kuqaia* were originally defined from the Mesozoic of China based on surface ornamentation: *Kuqaia quadrata*, characterized by “checked ornamentation”, *K*. *concentrica*, having “concentrical ridges thicker than radial ones”, and *K*. *radiata*, with “well-developed radial ridges” ([18], pp. 74–75). Two additional species were subsequently established: *K*. *yangii*, characterized by “concentric ridges on dorsum and venter and radial ridges on lateral sides”, and *K*. *yanqiensis*, having a “weakly ornamented or smooth shell surface” ([25], p. 303). *Aneules cucuma* was also transferred to *Kuqaia* [25]; however, identification of this species is problematic owing to the discrepancy between the illustration of the holotype and its description ([19]; Fig 4).

Although several species have been described (Fig 4), *Kuqaia quadrata* has been applied in a broad sense to all *Kuqaia* specimens recorded from the Norwegian offshore Jurassic strata based on the ornamentation in that population varying according to the contrasting degree and angle of compaction, and the quality of preservation [28]. Although some intraspecific variation is expected in the ornamentation of ephippia, we assign *Kuqaia* specimens to several discrete species, since there are clear visible differences among specimens regarding the pattern and robustness of shell surface ornamentation.

Among other acid-resistant mesofossil groups, the putative seed membrane *Chrysotheca diskoensis* Miner, 1935, with “oblong-ovate” body shape, bearing “3–6 plicate [sic] to the base, sessile or apparently short stalked” ornamentation on the body ([35], p. 590), has superficial morphological similarities to *Kuqaia concentrica* in bearing a predominance of concentric ridges. A later record of *Chrysotheca diskoensis* possesses transverse, although very thin and indistinct, ridge-like structures ([36], fig 36), which are somewhat similar to the ornament of *Kuqaia quadrata* but they encompass much larger lumina. Specimens of the dispersed seed membrane *Spermatites reticulatus* Kutluk & Hills, 2017 are also similar to *Kuqaia quadrata* in their possession of a raised reticulum/chequered ridges forming square and rectangular lumina, and some have a short stalk at the base of the main body [37]. However, *Spermatites* has radial symmetry and likely represents the megaspore membrane or inner testa coat of a seed-plant.

*Kuqaia scanicus* sp. nov.

Figs 2 and 3

*Derivation of name*. The specific epithet *scanicus* (Latin) refers to the discovery of this taxon in the southernmost province of Sweden, Scania (Skåne).

*Holotype*. NRM X12700 (Figs 2 and 3).

*Material*. Only the holotype, lacking the caudal end, is available.

*Type stratum and age*. Katslösa Member, Rya Formation; Pliensbachian.

*Diagnosis*. A *Kuqaia* bearing long peduncles on the lateral sides of the postventral margin.

*Description*. Shell reniform in lateral view; elliptical in dorso-ventral view. Concentric ridges weakly defined on the posterior half, conspicuous in the central part represented by *c*. 10 ridges, indistinct towards the ventral, dorsal, and the anterior parts. Radial ridges, well-developed on the anterior half with *c*. 19 ridges, becoming less well defined in the central part, and indistinct towards the posterior. Concentric and radial ridges are never strongly raised. Collar-shaped postventral margin is poorly preserved. Two long peduncles initiate from the lateral part of the postventral margin, are thicker on the posterior side, pointed at the terminus, *c*. 1200 μm long, and *c*. 35 μm wide at the posterior side. Peduncles bear weak, parallel and transverse ridges that become indistinct towards the tip. Back inconspicuous. Caudal spine damaged.

*Comparison*. This species is distinguished from all other representatives in the genus based on its possession of long peduncles.

*Dimensions*. Length 443 μm; width 200 μm (one specimen).

*Occurrence unit and age*. 40.40–39.60 m, Kävlinge BH-928; Katslösa Member, Rya Formation; Pliensbachian.

*Remarks*. The caudal spine of *Kuqaia* was initially emphasized as a primary morphological feature of this fossil group [18]. However, this slender structure is susceptible to breakage. On the single specimen stored in NRM, the central part of the postventral side of the shell is lacking due to incomplete preservation (Figs 2A and 3E). Since the other morphological characteristics of this species fall into the diagnosis of *Kuqaia*, we attribute the Swedish specimen to this genus, and infer that the caudal spine has been removed by physical damage.

*Kuqaia concentrica* Li, 1993

Fig 5A–5E

**Fig 5:**
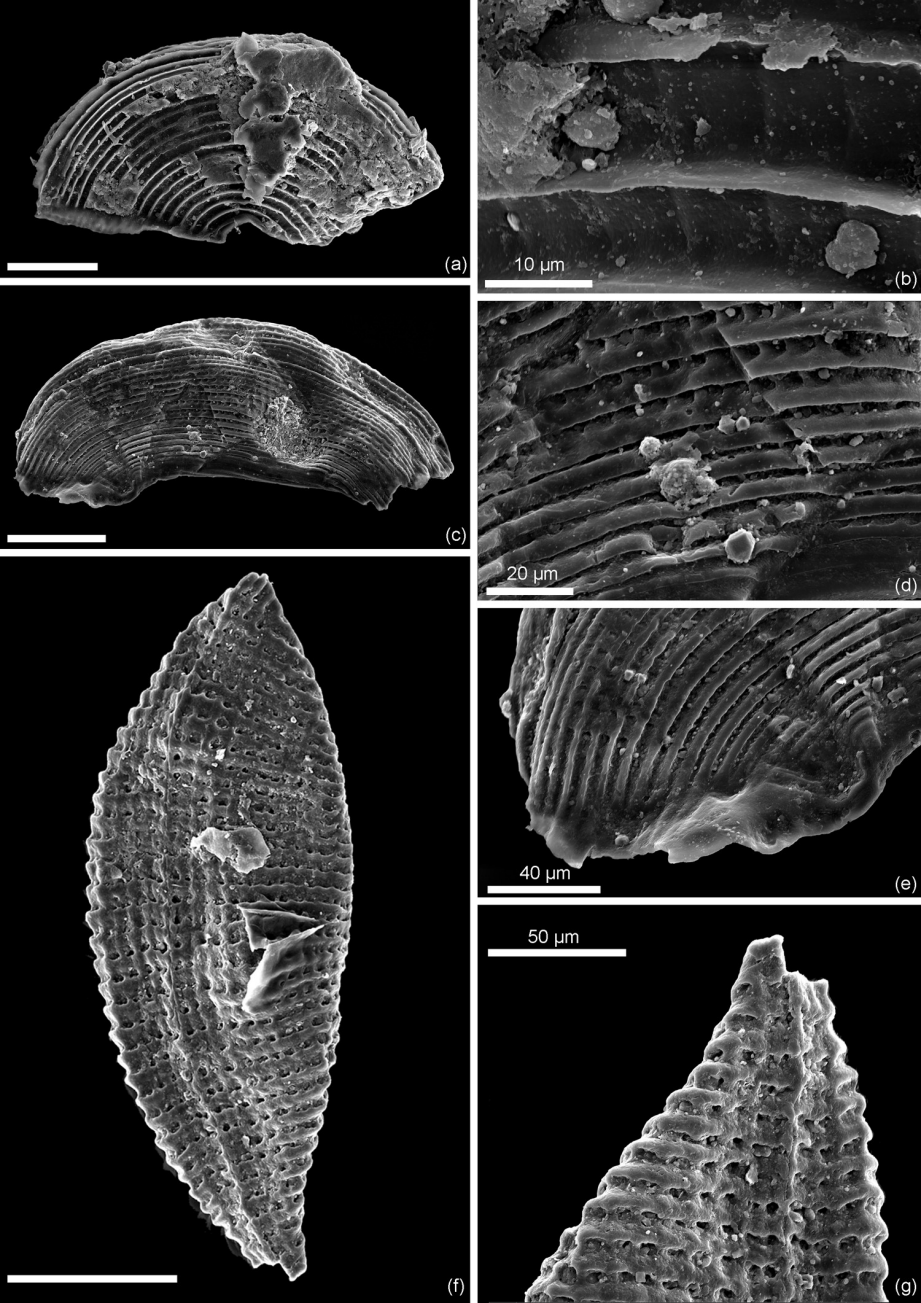
Scanning electron micrographs of *Kuqaia* species from the Pliensbachian of Skåne. (a)–(e) *Kuqaia concentrica* Li, 1993; (a) whole specimen (NRM X12701), and (b) enlargement of concentric ridges; Kävlinge BH-928 core depth 57.00–56.50 m; (c) whole specimen (NRM X12702); (d) and (e) enlargement of concentric ridges; Kävlinge BH-928 core depth 46.97–46.01 m. (f) and (g) *Kuqaia quadrata* Li, 1993; (f) whole specimen in oblique compression (NRM X12703), and (g) enlargement of intersecting concentric and radial ornament; Kävlinge BH-928 core depth 46.97–46.01 m. Scale bars = 100 μm, unless otherwise stated.

*Material*. NRM X12701; NRM X12702.

*Description*. Shell reniform in lateral view; elliptical in dorso-ventral view. Concentric ridges well-developed, 21–27 ridges on one valve, blade-shaped, or widened to 2–3.5 μm. Radial ridges weakly developed on the dorsal and lateral sides on one specimen. Collar-shaped margin poorly preserved on one specimen, *c*. 22 μm high. Back inconspicuous on these laterally preserved specimens. Caudal spine damaged.

*Comparison*. This species is distinguished from other species by its pronounced concentric ridges.

*Dimensions*. Length 444(451)457 μm; width 171(202)233 μm (two specimens).

*Remarks*. Two specimens are assigned to *Kuqaia concentrica* based on the ornamentation dominated by concentric ridges (Fig 5A–5E). One specimen is characterized by distinct blade-shaped concentric ridges on the shell, with weak radial ridges (Fig 5A and 5B). The other specimen is characterized by wider concentric ridges with modestly developed radial ridges in the dorsal and lateral parts (Fig 5D and 5E), resulting in a weakly developed pattern of chequered ridges similar to that in *Kuqaia quadrata* (Fig 5F and 5G). Nevertheless, the dominance of concentric ridges in the shell ornamentation of this specimen favours assignment to *Kuqaia concentrica*.

*Occurrence*, *unit and age*. 57.00–56.50 m and 46.97–46.07 m, Kävlinge BH-928; Katslösa Member, Rya Formation; Pliensbachian.

*Kuqaia quadrata* Li, 1993

**Fig 6:**
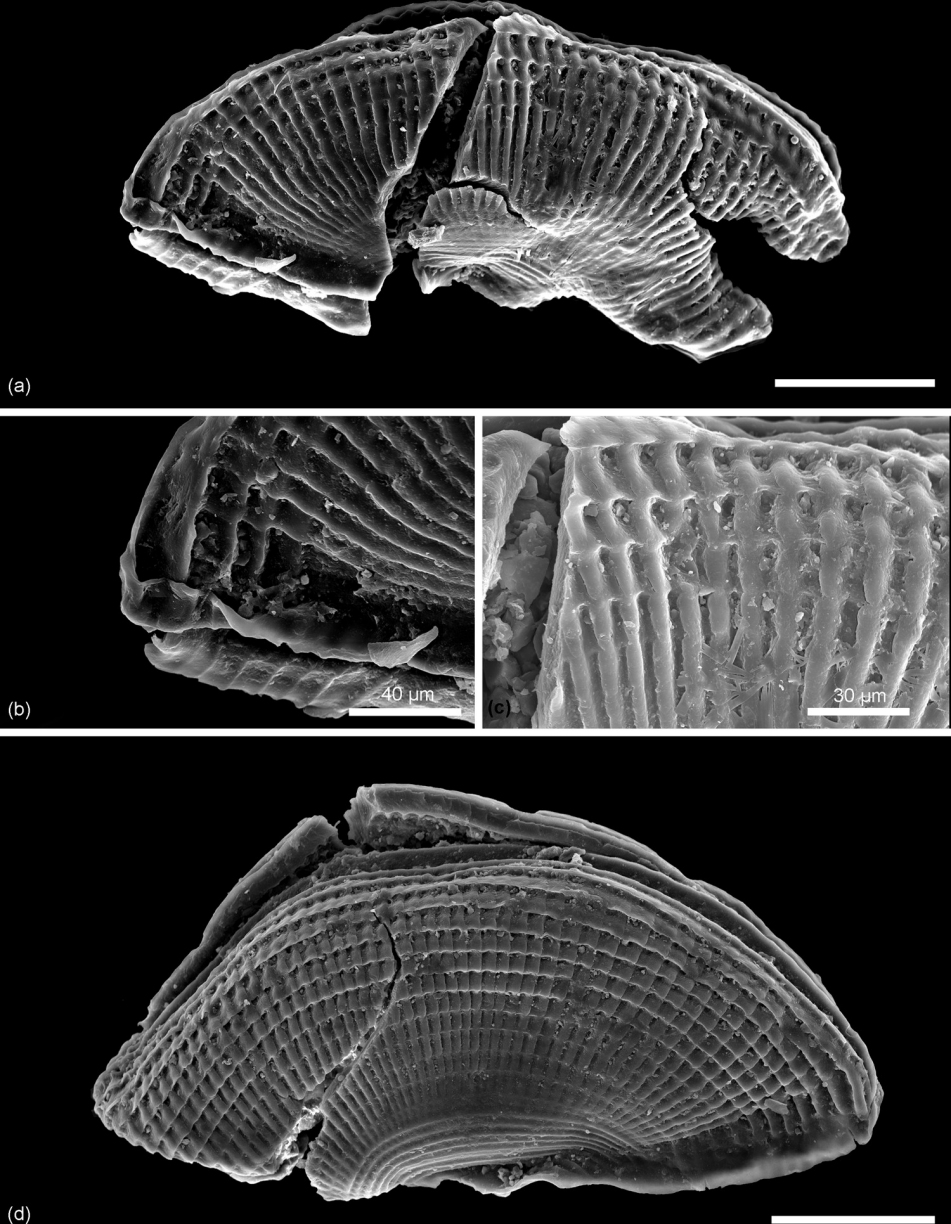
Scanning electron micrographs of *Kuqaia* species from the Pliensbachian of Skåne. *Kuqaia quadrata* Li, 1993; (a) one broken specimen (NRM X12704) showing intersecting concentric and more prominent radial ridges, (b) Collar-shaped postventral margin, and (c) enlargement of chequered ornament on the dorsal part; (d) lateral view of specimen (NRM X12705) showing typical intersecting concentric and radial ornament of equal prominence. All from Kävlinge BH-928 core depth 42.25–41.15 m. Scale bars = 100 μm, unless otherwise stated.

**Fig 7:**
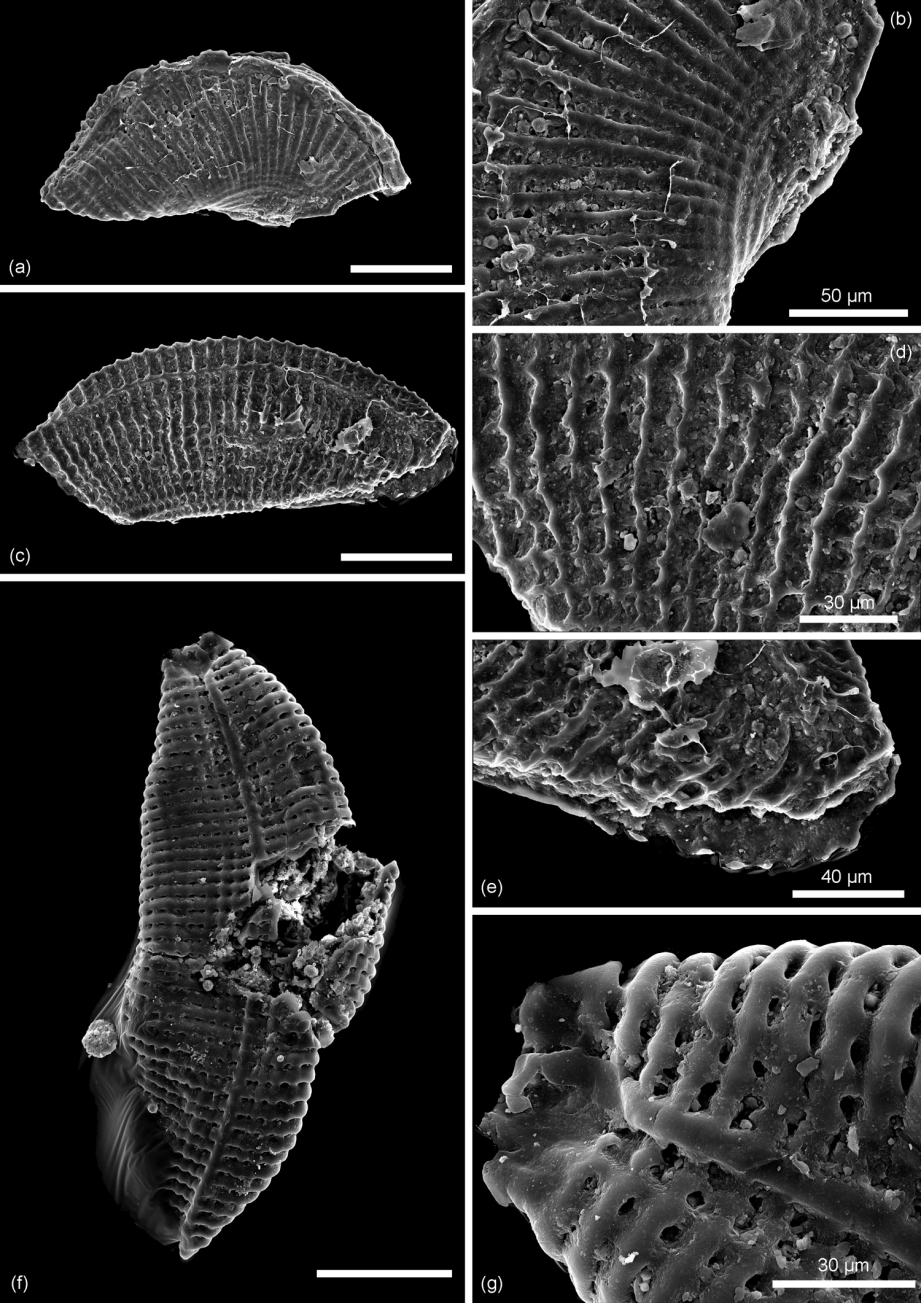
Scanning electron micrographs of *Kuqaia* species from the Pliensbachian of Skåne. (a)–(e) *Kuqaia radiata* Li, 1993; (a) whole specimen (NRM X12707), and (b) enlargement of radial ridges in lateral view, showing the prominence of radial ridges; (c) whole specimen (NRM X12708), (d) and (e) enlargements of radial ridges. (f) and (g) *Kuqaia quadrata* Li, 1993; (f) specimen (NRM X12706) in dorsal view showing the prominent intersecting ridged ornamentation and (g) enlargement of chequered ornament. All from Kävlinge BH-928 core depth 46.97–46.01 m. Scale bars = 100 μm, unless otherwise indicated.

Figs 5E, 5F, 6, 7F and 7G

*Material*. NRM X12703; NRM X12704; NRM X12705; NRM X12706.

*Description*. Shell reniform in lateral view; elliptical in dorso-ventral view. Shell surface covered with 34–51 radial and 21 concentric ridges (based on a specimen preserved in lateral view). Ridges are flat-crested, never blade-shaped; radial ridges 5–9 μm wide, concentric ridges 7–13 μm wide. Radial and concentric ridges are equally developed, forming a regular, rectangular mesh-like ornamentation. Collar-shaped margin preserved on one specimen, 22 μm high. Back conspicuous, slightly thicker than concentric ridges. Caudal spine damaged.

*Comparison*: This species is distinguished from other species based on the prominence of both concentric and radial ridges forming a chequered pattern.

*Dimensions*: Length 413(449)467 μm; width 150(186)240 μm (four specimens).

*Remarks*. One specimen (NRMX12704) with an ornamentation of regular rectangular meshes composed of flat interconnecting ridges (Fig 6A–6C), shows a predominance of concentric ridges at the venter and dorsum, and radial ridges in the lateral parts, possibly representing an intermediate ornamentation between *Kuqaia yangii* Cui *et al*., 2004 and *K*. *quadrata*. However, this specimen is broken, and the concentric ridges at the venter are inconspicuous; we tentatively identify it as *Kuqaia quadrata*.

*Occurrence*, *unit and age*. 46.97–46.07 m, 42.25–41.15 m, Kavlinge BH-928; Katslösa Member, Rya Formation; Pliensbachian.

*Kuqaia radiata* Li, 1993

Fig 7A–7E

*Material*. NRM X12707; NRM X12708.

*Description*. Shell reniform in lateral view; elliptical in dorso-ventral view. Radial ridges well-developed; 31–33 in number, flat-crested or blade-shaped, 3–7 μm wide. Concentric ridges generally weak, slightly more developed at venter and/or dorsum. On one specimen, concentric ridges incompletely developed at lateral sides, rarely reaching anterior and posterior. Back distinct. Collar-shaped margin and caudal spine are damaged.

*Comparison*. This species is distinguished from others by the predominance of radial ridges.

*Dimensions*. Length 365(383)400 μm; width 160(164)167 μm (two specimens).

*Remarks*. One specimen with both radial and concentric ridges forms a partially reticulate ornamentation (Fig 7C–7E). However, the shell surface is unambiguously dominated by radial ridges, thus favouring assignment to *Kuqaia radiata*.

*Occurrence*, *unit and age*. 46.97–46.07 m, Kävlinge BH-928; Katslösa Member, Rya Formation; Pliensbachian.

### Geographic distribution, geological range and abundance pattern

**Fig 8:**
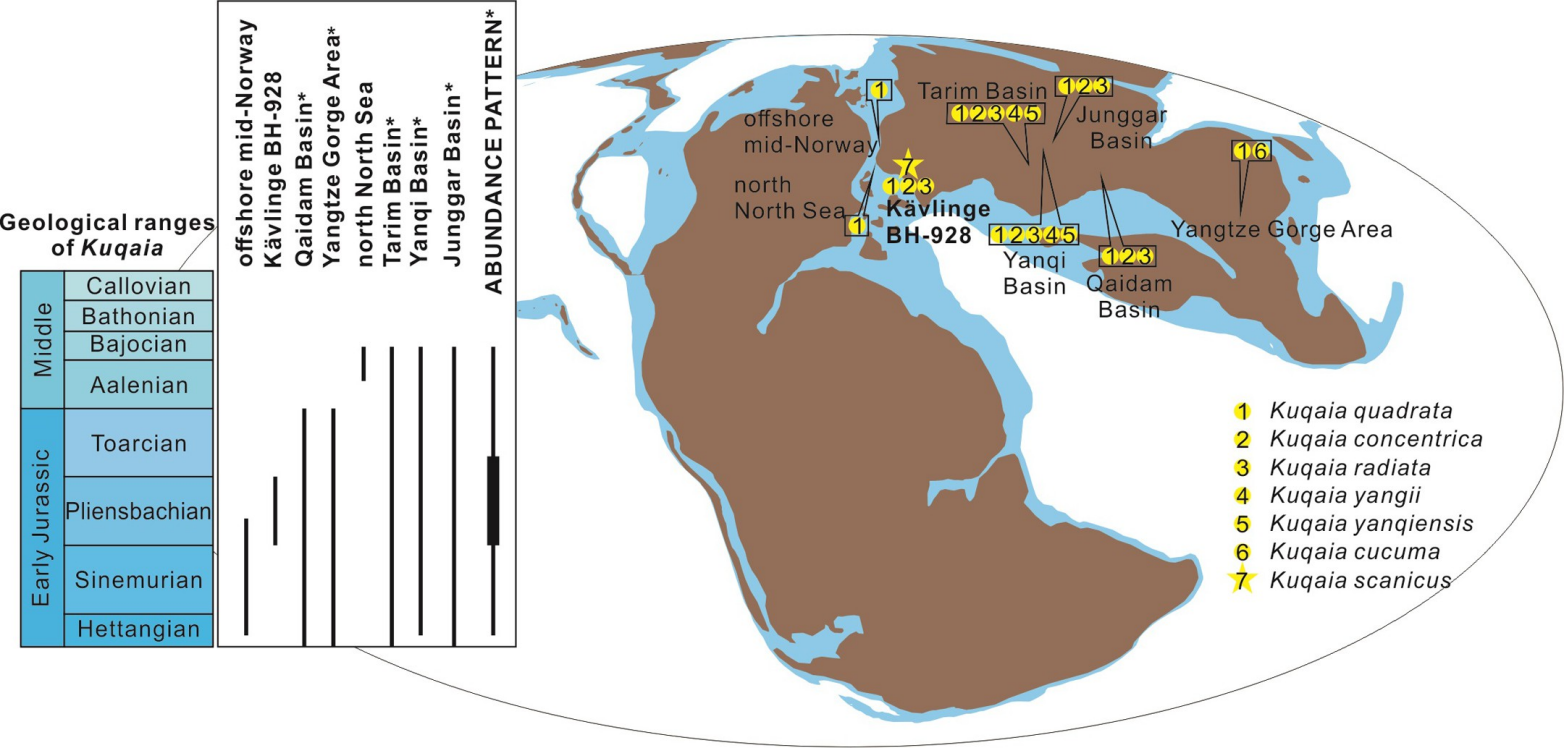
Geological ranges, palaeogeographic distributions, and apparent abundance pattern of *Kuqaia*. The base and top of *Kuqaia* stratigraphic ranges from each starred locality were tentatively calibrated based on material from offshore mid-Norway and the northern North Sea. The palaeogeographic map was modified after [38] under the Creative Commons Attribution 4.0 International License.

*Kuqaia* is recorded sporadically across Laurasia including, from east to the west, the Yangtze Gorge Area, Qaidam Basin, Yanqi Basin, Junggar Basin and Tarim Basin in China, southern Sweden, offshore central Norway, and the northern North Sea (Fig 8). Owing to their wide distribution and, until recently, uncertain systematic placement, we suspect that many examples of this genus have gone unreported in surveys of microfossil assemblages, or have been included as miscellaneous organic remains under categories such as acritarchs [22]. Examples referable to *Kuqaia quadrata*, *K*. *concentrica* and *K*. *radiata* are widely distributed in both eastern and western Laurasia [18, 26–29]. *Kuqaia yangii* and *K*. *yanqiensis* were documented in the Yanqi and Tarim basins in western China [25, 27], whereas *K*. *cucuma* is confined to the Yangtze Gorge Area [19]. Thus far, *K*. *scanicus* is known only from southern Sweden (this study).

The geological ranges of these fossils have been explored at generic level ([22, 27]; Fig 8), since species of *Kuqaia*, especially the widely distributed *Kuqaia quadrata*, *K*. *concentrica* and *K*. *radiata*, are normally associated in previously studied samples [18, 22, 24–27].

The oldest records of *Kuqaia* are from Hettangian strata of offshore Norway [28] where they are encountered in continental, freshwater deposits. The genus was not found in Triassic strata of that region. This accords with the records from the Yanqi Basin, where *Kuqaia* occurs slightly above the basal Jurassic strata [25]. The youngest occurrences of *Kuqaia* are in lower Middle Jurassic strata based on records from various sections in the Tarim, Yanqi, Junggar and Qaidam basins [23, 25, 26, 39]. The youngest occurrence of *Kuqaia* is in the northern North Sea succession within lower Bajocian strata [29]. Overall, *Kuqaia* is an index taxon of Lower to lower Middle Jurassic (Hettangian–Bajocian) strata.

A general change in the abundance of *Kuqaia* has been proposed based on quantitative data from stratigraphic successions in the Tarim, Yanqi and Junggar basins, northwestern China ([22, 25, 39]; Fig 8). *Kuqaia* appears in the Ahe and Badaowan formations (lower Lower Jurassic) in low abundances, is more common in the overlying Yangxia and Sangonghe formations (upper Lower Jurassic), and decreases in the succeeding Kezilenur and Xishanyao formations (lower Middle Jurassic). However, these quantitative trends are equivocal given that yields of *Kuqaia*-sized fossils, e.g., mesofossils, vary greatly between samples (e.g., from 1 to more than 200 specimens) as a consequence of taphonomic sorting.

### The biological affinities of *Kuqaia* and their paleoecology

*Kuqaia* was first defined as belonging to an unknown palynomorph group owing to its resistance to HF during palynological processing [18] and later suggested to represent a megaspore or fragment thereof [20]. It has also been proposed that *Kuqaia* represents a group of gastropods [22].

*Kuqaia* ‘shells’ are typically 300–500 μm long, placing them in the typical size range of extant rotifers (100–500 μm long). The lorica (external cuticle) of rotifers is composed of scleroproteins, is resistant to acids, and some taxa bear elaborate ornamentation along with anterior and posterior spines and other appendages [40, 41]. Acid-resistance confers generally good preservational potential for those rotifers with a well-developed lorica, and this group has a fairly extensive, though under-appreciated, fossil record [42–45]. However, the lorica of rotifers is composed of several plates, is generally box- or tube-shaped, ornamentation does not generally include concentric ridges, and the posterior appendage (foot) is typically segmented [46].

**Fig 9:**
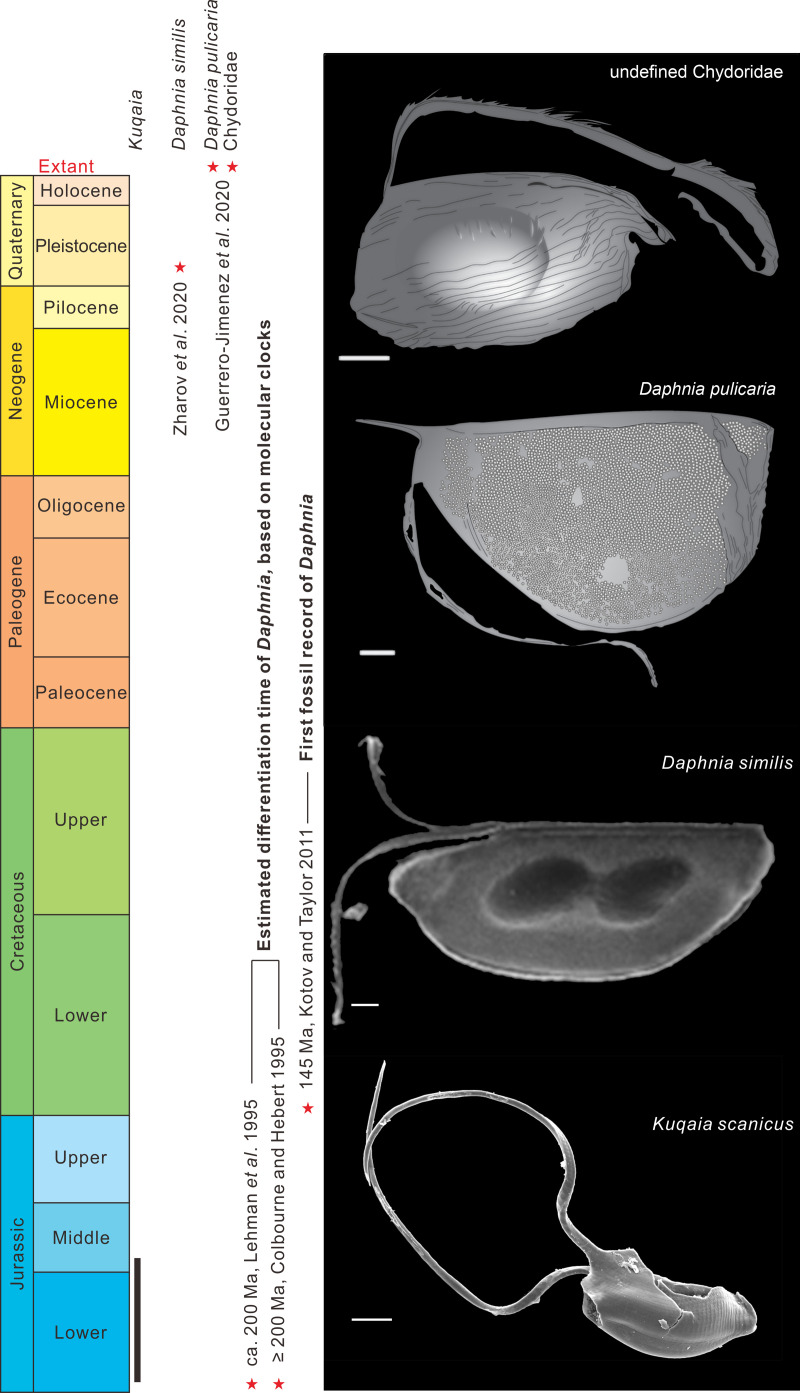
*Kuqaia scanicus* sp. nov. and morphologically similar fossils of *Daphnia* ephippia. The geological ages, the estimated dates of differentiation of the *Daphnia* lineage, and the age of the oldest fossil record of *Daphnia*, are plotted. Scale bars = 100 μm. SEM image of *Daphnia similis* used with permission from [48]; line drawings of *Daphnia pulicaria* and ‘undefined Chyoridae’ compiled by the authors and interpreted from images in [34].

Although most are slightly smaller, at <500 μm long, *Kuqaia* shells have morphological similarities to the ephippia (encasings protecting the dormant embryos) of extant cladocerans (Crustacea: Branchiopoda: Cladocera/Diplostraca), at 400–2500 μm long ([34, 47, 48]; Fig 9). Apart from their microscopic size, *Kuqaia* shares with cladoceran ephippia, a HF-resistant wall, roughly semicircular shell shape and elongate posterior appendages.

In particular, the presence of caudal spines in *Kuqaia* represents a striking similarity to the ephippia of extant *Daphnia*. An extant cladoceran specimen, possibly attributable to *Leydigia* (Chydoridae) based on the long setae at the ventral margin [49] (Fig 9), produces ephippia [34] that are similar to *Kuqaia scanicus* as evidenced by the presence of the markedly elongate appendages, almost twice the length of the shell body. The presence of peduncles in *Kuqaia scanicus* is strongly similar to the architecture of extant *Daphnia pulicaria* ephippia ([34]; Fig 9). The presence of peduncles also links *Kuqaia scanicus* to the fossil ephippia of *Daphnia*; e.g., it has similarities to the ephippia of Pleistocence *D*. *similis* ([48]; Fig 9), except that *K*. *scanicus* bears two longer (with respect to the shell body) peduncles on the lateral part of the postventral end, whereas the two shorter peduncles of *D*. *similis* originate from the central part of the postventral end (Fig 9).

Although *K*. *scanicus* has similar morphological features to the ephippia of several types of extant and fossil cladocerans (Fig 9), *Kuqaia* is distinguishable from the majority of extant and fossil ephippia of *Daphnia* in lacking any swellings or other morphological features that typically demarcate the location of the enclosed single or paired loculi ([34, 47, 48, 50–54]; Fig 9). Anomopod ephippia were described from Lower Cretaceous strata at the Khutel-Khara locality, Mongolia [50]. These bear one loculus on the lateral side of the ephippia. Similar ephippia with a single-egg/embryo loculus were recorded in the Lower Cretaceous Jehol Biota, northeastern China [51]. *Daphnia* fossils, including their associated ephippia, have also been documented from Cenozoic deposits in Germany [52]. These bear two-egg loculi located obliquely or at acute angles to the dorsal margin. Such features are not apparent on *Kuqaia*. Other late Cenozoic *Daphnia* ephippia also bear two-egg loculi [53–55]. Loculus orientation can be important in distinguishing ephippia of various cladoceran taxa. However, the locule position is not always evident on the exterior of ephippia and, depending on environmental conditions, some extant species of *Daphnia* produce between 35 and 70% of ephippia lacking an obvious locule [56]. The lack of strongly varied shell surface ornamentation in *Kuqaia* is suggestive of a morphologically simple, archaic group of cladocerans, possibly on the stem group lineage of *Daphnia*.

Comparisons with pre-Cenozoic forms are hindered by the sparse fossil record of Cladocerans. The oldest putative cladoceran fossils are those recorded from the Devonian Rhynie and Windyfield cherts and an *ex situ* Carboniferous cobble from Yorkshire, UK [57, 58]. However, attribution of the Paleozoic fossils to Cladocera has been questioned [59], and no ephippia were identified with these ‘cladocerans’. Sparse examples of this group also derive from Mesozoic strata from various parts of the world, but substantial gaps in the fossil record constrain our understanding of the evolution and diversification of cladocerans through time [59]. Jurassic ephippia [60] are sufficiently distinctive to be assigned to extant genera, so the origins of modern groups likely extend back at least to the early Mesozoic [61].

Shell surface ornamentation is variable (laevigate, granulate, ridged and reticulate) on the ephippia of *Daphnia*, such that subgenera can be recognized based on fossil ephippial morphology. The shell surfaces of *Kuqaia* are smooth and ridged based on studies thus far. Extant *Daphinia pulicaria* produces ephippia with a granulate surface ornamentation ([34]; Fig 9). Other Mesozoic anomopod ephippia differ from *Kuqaia* in having tuberculate shell surfaces [50]. Ephippia of the late Cenozoic *Daphnia pulex* group bear reticulate shell surfaces with setae at the ventral margin [53].

Intriguingly, molecular dating of the divergence of the *Daphnia* lineage from other cladocerans is inferred to be *c*. 200 or ≥200 Ma ([62, 63]; Fig 9). By contrast, the earliest known fossil records of *Daphnia* are from the Jurassic–Cretaceous boundary (145 Ma, [64]; Fig 9). Significantly, the first appearance of *Kuqaia* coincides with the estimated divergence of *Daphnia*, supporting the hypothesis that *Kuqaia* may represent a stem group of the *Daphnia* lineage.

The geological distribution of *Kuqaia* indicates exclusively continental, freshwater to brackish lagoonal environments in line with extant Cladocera [65]. As the global climate during the Early Jurassic was warmer than present, based on fossil records and palaeo-CO_2_ reconstructions estimating atmospheric concentrations [66], we argue that the resting eggs would have produced during dry-season intervals in middle latitudes, rather than as a result of winter cooling. The dark colour of the studied specimens suggests the presence of protective melanin [67], which might indicate that they inhabited clear-water lakes.

Although resistance to strong acids suggests a sporopollenin or chitinous/pseudochitinous composition like most other palynomorphs [68], the chemistry of *Kuqaia* fossils is not known. Similarly, the composition of extant cladoceran ephippia shells has been little studied but is generally assumed to be chitinous with a high proportion of melanin [59, 69]. An alternative is that these highly durable resting cases containing eggs or embryos are composed of a scleroprotein similar to the egg-bearing cocoons of leeches [70, 71], earthworms [72], and the lorica of rotifers [73]. Although the morphological similarities with cladocerans (Diplostraca), such as *Daphnia* are strong, future chemical analysis of *Kuqaia* mesofossils and extant cladocerans is clearly warranted, since this might help better resolve their biological affiliation.

## Conclusions

The new mesofossil species, *Kuqaia scanicus*, is instituted based on an exceptionally preserved specimen recovered from the drill core Kävlinge BH-928, in southern Sweden. The spatial and temporal distributions of *Kuqaia* species indicate a Laurasian (middle northern latitudes of Pangea) distribution for the genus and indicate its potential use as a biostratigraphic marker for Hettangian–Bajocian strata of this region. Based on morphological evidence, *Kuqaia* is most similar to the resting egg/embryo cases (ephippia) of cladocerans (diplostracans), and likely represents an early stem-group record of the planktonic crustacean *Daphnia* lineage. We recommend greater attention be given to chemical analyses of the acid-resistant eggs and egg cases of fossil and extant invertebrates to improve resolution of the biological affiliations of enigmatic mesofossil groups, such as *Kuqaia*.

Given their broad geographic distribution, we suspect that cladoceran ephippia are much more abundant in the fossil record than previously assumed. The scarcity of past records of this taxon probably relates to their size, composition and lack of targeted searches. At 300–700 μm long, *Kuqaia* remains are generally too large to be recovered in palynological residues but too small to be recognized in macrofossil assemblages. *Kuqaia* specimens are typically extracted from siliciclastic rocks via hydrofluoric-acid bulk dissolution (a process that usually destroys non-palynomorph microfossils), and most are recovered from residues specifically targeting organic mesofossils, such as megaspores. Once concerted exploration for *Kuqaia* and other cladoceran remains has been carried out, this group offers considerable value for reconstructing food-web functionality of ancient freshwater ecosystems since these organisms are among the few pelagic primary consumers in these communities that have high potential for fossilization.
